# Lytic Transcriptome Dataset of Varicella Zoster Virus Generated by Long-Read Sequencing

**DOI:** 10.3389/fgene.2018.00460

**Published:** 2018-10-16

**Authors:** Dóra Tombácz, István Prazsák, Norbert Moldován, Attila Szűcs, Zsolt Boldogkői

**Affiliations:** Department of Medical Biology, Faculty of Medicine, University of Szeged, Szeged, Hungary

**Keywords:** varicella zoster virus, long-read sequencing, transcriptome, full-length sequencing, nanopore-based sequencing, Oxford Nanopore Technologies

## Introduction

Varicella zoster virus (VZV) belongs to the *Alphaherpesvirinae* subfamily of the *Herpesviridae* family. It is the etiological agent of chickenpox (varicella) caused by primary infection and shingles (zoster), which is due to reactivation of the virus from latency (Kennedy, 2002). Many countries have adopted recommendations for routine immunization of children and susceptible adults against VZV. The VZV virion is composed of an icosahedral nucleocapsid surrounded by a tegument layer, which is covered by an envelope derived from the host cell membrane with incorporated viral glycoproteins (Maresova et al., 2005). The genome of VZV consists of a linear double-stranded DNA molecule and is approximately 125 kbp in size, which contains more than 70 annotated open reading frames (ORFs) (Tyler et al., 2007). The transcription of the virus is strictly regulated by cascade-like processes. First, the immediate-early (IE) transcripts are expressed, which is then followed by the expression of the early (E), and then the late (L) kinetic classes of transcripts (Reichelt et al., 2009). The IE ORF62 gene of VZV encodes the major transactivator, which controls the expression of other viral genes. The viral E genes encode proteins that are used in DNA replication, while L genes code for the structural elements of the virus.

High-throughput short-read sequencing (SRS) techniques have revolutionized transcriptome research (Delseny et al., 2010). These techniques have also been utilized in the investigation of herpesvirus gene expression (e.g., Chambers et al., 1999; Ebrahimi et al., 2003; Baird et al., 2014; Oláh et al., 2015). However, the SRS approach has severe limitations in comparison to long-read sequencing (LRS), including Pacific Biosciences (PacBio) and Oxford Nanopore Technologies (ONT) platforms. LRS techniques have been used before in transcriptome studies of the herpesviruses (O'Grady et al., 2016; Tombácz et al., 2016, 2017; Balázs et al., 2017a,b; Moldován et al., 2018). These studies uncovered a very complex transcriptome, which included the identification of a large number of novel RNA molecules and transcript isoforms (Tombácz et al., 2015, 2017; Balázs et al., 2017a). Moreover, an extended meshwork of overlaps between the transcripts was also detected by these studies (Tombácz et al., 2016; Moldován et al., 2018).

The presented data report is aimed toward providing a new, comprehensive transcript catalog of VZV using an LRS approach for the first time. In this study, we applied the ONT MinION device and various full-length cDNA sequencing protocols that capture the entire poly(A)-transcriptome of VZV.

## Value of the data

1. Varicella zoster virus (VZV) is world-wide distributed human pathogenic alphaherpesvirus.
2. No long-read sequencing (LRS) transcriptome data from VZV has been published thus far. Here, we provide a dataset on the lytic polyadenylated transcriptome of the VZV generated by LRS from Oxford Nanopore Technologies.
3. LRS approaches have been reported to be superior to other methods, including short-read sequencing in the detection of embedded RNA molecules, polycistronic transcripts and transcriptional overlaps, as well as in distinguishing between RNA isoforms including splice and transcript end variants. These data will be useful for analyzing the complexity of VZV transcriptome, for identifying novel transcripts and for comparing the ONT platform with other cDNA sequencing approaches.

## Data

In this work, the ONT MinION sequencing technique was used for the analysis of the genome-wide expression of VZV genes using the 1D cDNA sequencing protocol. For the detection of full-length transcripts, a modified version of this approach was also carried out, starting with Cap-selection of the RNA samples, using a so called “all-in-one” Cap-selection protocol, but this technique produced very short average read lengths (Table 1). We obtained the same poor result with pseudorabies virus (PRV; Moldován et al., 2018; Tombácz et al., 2018) and Herpes simplex virus−1 (HSV; submitted). In contrast, Cap-selection performed very well in the analysis of the transcriptome of a baculovirus (unpublished) and vaccinia virus (VACV, unpublished). From these results, it has become apparent that not the AT-content (VACV: 66%, VZV: 54%, HCMV: 42%, HSV: 32%, PRV: 27%) accounts for the short read length in the Cap-selected samples, but rather an unknown factor that prevents the completion of reverse transcription in only the alphaherpesviruses.

**Table 1:** Summary of the obtained reads and read qualities mapped to the VZV genome (Unique: duplicates have been removed; Duplicates: duplicate reads are included in the statistics).

	**1D cDNA**	**CAP seq**
Reads^A^	57888	509531
Average^B^	1470,14	426,92
Median^C^	1033	258
Average^D^	1399,43	307,11
SD^E^	993,11	148,02
Coverage^F^	648,68	1253,03
Del^G^	3,74	4,16
SD^H^	1,74	2,18
Ins^I^	3,48	2,63
SD^J^	1,67	2,12
MM^K^	5,41	4,75
SD^L^	2,14	2,4

A number of mapped reads;

B average of read lengths;

C median of the aligned reads;

D average of the aligned reads;

E SD of average;

F read coverage across the VZV genome;

G percentage of deletions;

H standard deviations;

I percentage of insertions;

J standard deviations;

K percentage of mismatches;

L *standard deviation values of mismatches*.

**Figure 1:**
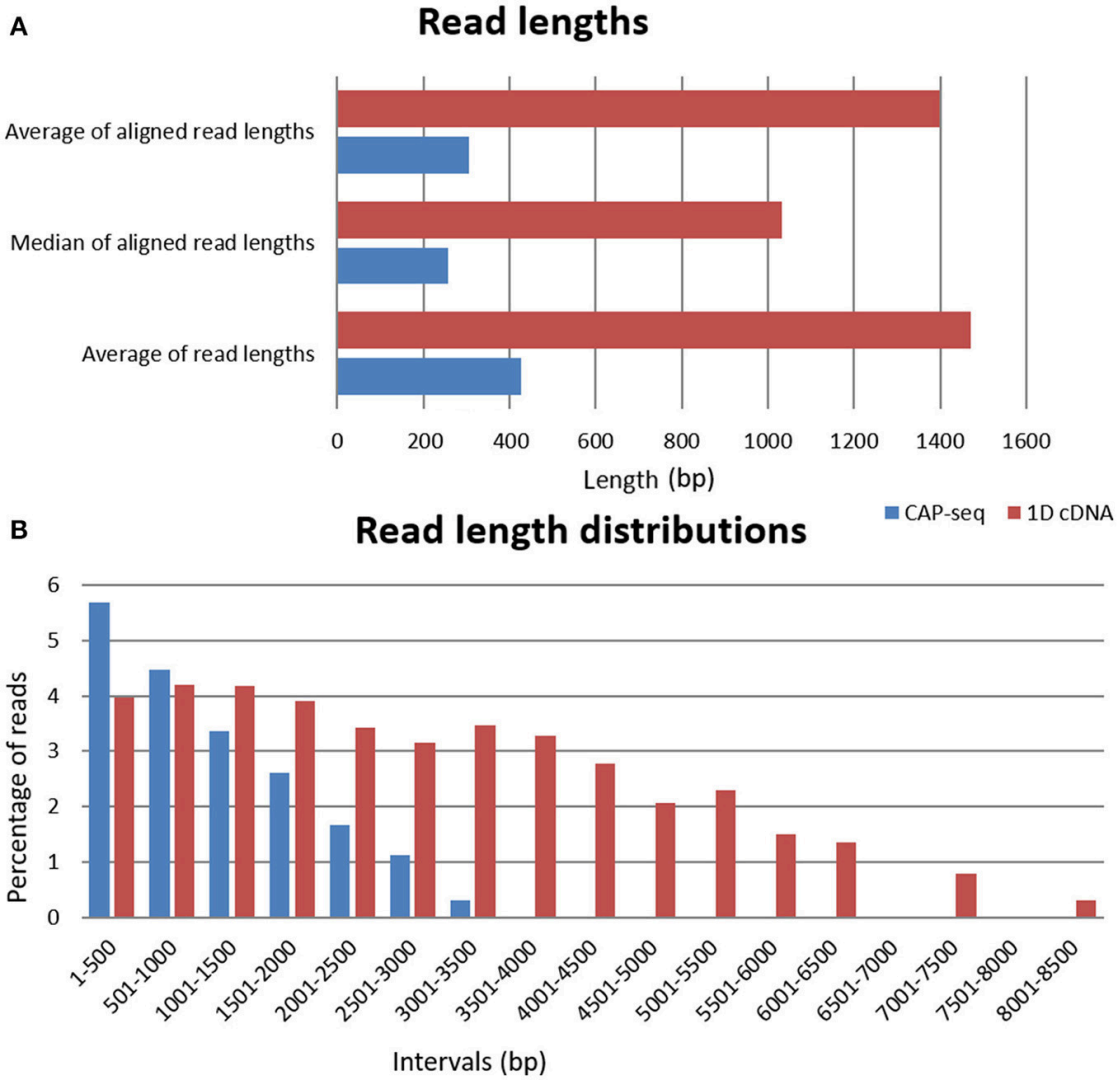
Read length and read length distributions. **(A)** The horizontal bar charts show the average read lengths of the two different libraries, as well as the median and the average read lengths mapped to the viral genome. **(B)** The average distribution of read lengths which align to the VZV genome; binned to 500bp intervals at log10 scale.

ONT 1D cDNA sequencing yielded 57,888 VZV-specific sequencing reads with an average coverage of 649, while the sequencing on Cap-selected samples resulted in 509,531 reads (1,253-fold coverage) mapped to the VZV genome (NC_001348.1). The average read-lengths for the cDNA sequencing and for the Cap-sequencing were 1,470 and 427bp, respectively (Table 1, Figure 1A).

ONT sequencing is able to read full-length RNA molecules, but it falls short in accuracy. due to its high-throughput workflow. Additionally, we used barcoding for better identification of the transcripts' ends. ONT sequencing is afflicted by sample degradation, which in general can be eliminated by using Cap-selection. Reads were sorted by the Albacore v. 2.0.1 according to their q-sore in two categories: pass and fail. Only the reads belong to the passed category were used in further analysis. Non-virus specific sequencing reads were filtered by mapping to the above mentioned viral genome using GMAP.

## Materials and methods

### Cell culture and virus growth

Human primary embryonic lung fibroblast cell line (MRC5) was obtained from the American Type Culture Collection and grown in Dulbecco's modified Eagle's minimal essential medium (Sigma-Aldrich, St. Louis, MO, USA) supplemented with 1% of an antibiotic/antimycotic (AB/AM) solution (Lonza, Walkersville, MD, USA) and 10% fetal bovine serum (GE Healthcare Life Sciences, Logan, UT, USA) at 37°C in a 5% CO_2_ atmosphere. The live attenuated OKA/Merck strain of varicella zoster virus (VZV) (MSD Pharma Hungary Kft., Budapest, Hungary) was cultured at 37°C in MRC5 cell line, and the cells were harvested by trypsinization, when the monolayers had displayed near 80% specific cytopathic changes. For sub-passage of the virus or for experiments, the VZV-infected whole cells were used to inoculate MRC5 cultures previously grown to full confluence at a ratio of one infected cell to ten uninfected cells. The cultures were then incubated at 37°C for 5 days.

### RNA purification

Total RNA was isolated from the infected cell using the NucleoSpin® RNA kit (Macherey-Nagel) as was previously described (Tombácz et al., 2016, 2017; Balázs et al., 2017a). Total RNA samples were handled by Ambion® TURBO DNA-free™ Kit (Thermo Fisher Scientific) to remove to potential gDNA contamination. For the 1D cDNA sequencing, the polyA(+) fraction was extracted from the total RNA samples by using the Qiagen Oligotex mRNA Mini Kit, following the “Spin Columns” protocol of the kit. Samples were quantified by Qubit 2.0 fluorimeter using the Qubit RNA BR and HS Assay Kits (Life Technologies) for the total RNA and PolyA(+)RNA measurement, respectively and then they were stored at −80°C until use.

### Generation of sequencing libraries

An ONT MinION device was used for the analysis of the full-length transcriptome profile of VZV. We applied two library preparation protocols. The 1D cDNA sequencing was carried out by using the 1D Strand switching cDNA by ligation protocol (Version: SSE_9011_v108_revS_18Oct2016). After the first End-prep step, a barcode (C11 barcode: ONT PCR Barcoding Kit 96; EXP-PBC096) was ligated to the cDNA samples following the relevant part of the 1D PCR barcoding (96) genomic DNA (SQK-LSK108) protocol, for better identification of the 5′ end of the reads. We also applied a Cap-selection method combined with the 1D protocol for the detection of the 5′-ends of the transcripts. The TeloPrime Full-Length cDNA Amplification Kit (Lexogen) was used for the cDNA preparation. Total RNA was used for the reverse transcription (RT). The sample was mixed with RT buffer and a specific primer (both are part of the kit). The reaction started with incubation at 70°C for 30 s, which was then followed by a 1 min step at 37°C. The reverse transcriptase enzyme and the additional reagents (components of the kit) were mixed with the sample and then the reaction was contained at 37°C for 2 min. The next incubation step of the RT was carried out at 46°C for 50 min. The sample was purified by using the kit's Silica columns. The double-strand (ds) specific ligase enzyme (Lexogen kit) was used to join the adapter to the cDNA. The ligation was done at 25°C overnight, then the sample was purified using the silica membranes of the kit. The dscDNAs were generated by using the Enzyme Mix and the Second-Strand Mix (Lexogen kit). The cDNA generation was carried out in a Veriti thermal cycler, applying the following protocol: 98°C for 90 s, 62°C for 60 s, 72°C for 5 min (16 cycles), hold at 25°C.The library production from the dsDNA was based on the 1D protocol; the end-repair and the 1D adapter ligation (NEBNext End repair / dA-tailing Module NEB Blunt/TA Ligase Master Mix) steps were carried out by using the 1D protocol and kit. The ready libraries were run on an ONT R9.4 SpotON Flow Cells.

The concentration of both the dscDNAs, as well as the libraries was detected by using Qubit 2.0 and Qubit dsDNA HS quantitation assay (Life Technologies).

### Mapping, data processing, and statistics

The ONT's Albacore software v2.0.1 was used for base calling. These sequencing reads were aligned using the GMAP (Wu and Watanabe, 2005) version 2017-09-30 with default setting to the genome NC_001348. Statistics about the read quality, such as insertions, deletions, and mismatches, as well as the coverages can be found in Table 1. In house scripts were used to obtain the quality information presented in this data report (Github, doi:10.5281/zenodo.1034511). The basic statistic data about the FASTQ files are shown in Supplementary Table 1. The FASTQ analysis was carried out using the ea-utils package (Aronesty, 2011). This toolkit includes programs for calculating sequencing and alignment statistics, demultiplexing, and variant calling. The fastq-stats program from this software package was used to obtain data base quality scores, and additional basic information such as base composition, base count, read lengths. FastQC version 0.11.5 was used to generate quality reports (deposited in Figshare; https://figshare.com/articles/Varicella_Zoster_FastQC_data/7016372, Supplementary Table 2). The read length distribution of the samples is visualized in Figure 1B.

## Data availability

The sequencing data and the transcriptome assembly have been uploaded to the European Nucleotide Archive under the project accession number: PRJEB25401. The FASTQ and binary alignment (BAM) files have also been uploaded for each experiment to facilitate the usage of the dataset. The FASTQ files can be aligned to any reference genome, while the BAM files contain reads already mapped to the NC_001348.1. Sample SAMEA104667607, Run accession ERR2366789 is the polyA selected experiment, while sample SAMEA104667608, run accession ERR2366790 is the CAP selected experiment.

## Data availability statement

The datasets generated for this study can be found in the European Nucleotide ArchivePRJEB25401 [https://www.ebi.ac.uk/ena/data/search?query=PRJEB25401]; named as “Long-read Sequencing Dataset of Varicella Zoster Virus.”

## Author contributions

DT carried out ONT sequencing, data analysis, and drafted the manuscript. IP take part in RNA purification, sequencing, and data analysis. NM participated in data analysis. AS carried out bioinformatics analysis. ZB conceived and designed the experiments, and wrote the manuscript. All authors read and approved the final paper.

## Conflict of interest statement

The authors declare that the research was conducted in the absence of any commercial or financial relationships that could be construed as a potential conflict of interest.

## Supplementary material

Supplementary Table 1 Basic statistics of the reads. ^A^number of reads in the given file; ^B^mean of the read lengths in given FASTQ; ^C^standard deviation; ^*D*^Phred scale used (Phred scale also known as the quality or Q scores of a base, which are often represented as ASCII characters. Tables converting between integer Q scores, ASCII characters and error probabilities are shown in the upper table in the following website: https://www.drive5.com/usearch/manual/quality_score.html. The ASCII_BASE 33 is now almost universally used for short – and long-read sequencing techniques); ^E^Number of reads used to generate duplicate read statistics (the total read count of a given sample was used for the duplicate analysis); ^F^base Quality min; ^G^base Quality max; ^H^base Quality mean; ^I^base Quality SD; ^J^ A content (%); ^K^C content (%); ^L^G content (%); ^M^T content (%); ^N^total number of bases; (Unique: duplicates have been removed; Duplicates: duplicate reads are included in the statistics).

Supplementary Table 2 This table summarizes the type of samples and the related FastQC file names, which have been deposited to the Figshare.
